# Family Functioning, Illness-Related Self-Regulation Processes, and Clinical Outcomes in Major Depression: A Prospective Study in Greece

**DOI:** 10.3390/healthcare11222938

**Published:** 2023-11-10

**Authors:** Katerina Koutra, Georgios Mavroeides, Maria Basta, Alexandros N. Vgontzas

**Affiliations:** 1 Department of Psychology, School of Social Sciences, University of Crete, 74100 Rethymnon, Crete, Greece; mavroeides@gmail.com; 2 Department of Psychiatry & Behavioral Sciences, Faculty of Medicine, University of Crete, 71003 Heraklion, Crete, Greece; mpasta@uoc.gr; 3 Mobile Mental Health Unit, University Hospital of Heraklion, University of Crete, 71003 Heraklion, Crete, Greece; avgontzas@pennstatehealth.psu.edu; 4 Sleep Research & Treatment Center, Department of Psychiatry & Behavioral Health, Penn State College of Medicine, Hershey, PA 17033, USA

**Keywords:** coping strategies, family functioning, illness representations, major depressive disorder, symptom severity, suicide risk

## Abstract

Major depressive disorder (MDD) is a common, seriously impairing, and often recurrent mental disorder. Based on the predictions of the Circumplex Model of Marital and Family Systems and the Common-Sense Self-Regulation Model, the aim of the present prospective study is to examine the predictive value of clinical outcomes of a process model in which associations between perceived family functioning and patient’s clinical outcomes (i.e., symptom severity and suicide risk) are mediated by illness representations and coping strategies. A total of 113 patients with a clinical diagnosis of MDD (16.8% males and 83.2% females) aged 47.25 ± 13.98 years and recruited from the outpatient department and the mobile mental health unit of the Psychiatric Clinic of the University Hospital of Heraklion in Crete, Greece, and from a Greek online depression peer-support group participated in the study. Family functioning was assessed in terms of cohesion and flexibility (Family Adaptability and Cohesion Evaluation Scales IV) at baseline. Illness representations (Illness Perception Questionnaire–Mental Health) and coping strategies (Brief Cope Orientation to Problems Experienced) were measured about five months later (5.04 ± 1.16 months). Symptom severity (Beck Depression Inventory) and suicidality (Risk Assessment Suicidality Scale) were measured about 10 months after the baseline assessment (9.56 ± 2.52 months). The results indicated that representations about MDD impact and symptom severity serially mediated the association between family cohesion and suicide risk in MDD. Furthermore, family cohesion was found to be linked with maladaptive coping through MDD impact representations. Family-based psychotherapeutic interventions specifically designed to target unhealthy family functioning, along with negative illness perceptions and dysfunctional coping, could be further developed and explored as adjunctive therapy to standard treatment in MDD.

## 1. Introduction

### 1.1. Major Depressive Disorder

Major depressive disorder (MDD) is a common, seriously impairing, and often recurrent mental disorder [1,2]. In March of 2017, MDD was declared by the World Health Organization to be the illness with the greatest burden of disease in the world [3]. Despite decades of widespread intervention, research, and public awareness efforts, prevalence rates of MDD remain increasing [4]. Although effective biological and psychosocial interventions are available, clinically meaningful adverse outcomes for people with a diagnosis of MDD are still high [5]. Suicidality remains one of the most serious outcomes of MDD, and, according to two recent meta-analyses, prevalence rates for suicidal ideation and suicide attempts in MDD were 37.7% and 31.0%, respectively [6,7], while MDD accounts for up to 87% of completed suicides [8]. The reasons for MDD clinical adverse outcomes remaining that high are not fully understood. Identifying the factors that lie behind these variations in response to MDD is extremely important in order to improve outcomes for individuals and their families.

### 1.2. Family Functioning in MDD

The impact of MDD is not limited to the individual but also affects their family members. Special attention has been paid to the spouses/partners of individuals with MDD who often have a caregiving role, especially in societies characterized by tight-knit bonds and interactions, such as Greece. However, there have been relatively few studies examining aspects of family functioning (i.e., cohesion, conflicts, etc.) in MDD. These studies have shown that families of patients with MDD may have functional deficits in family functioning as compared to control families [9,10,11] or other clinical groups, such as individuals with bipolar disorder [12,13]. Research also suggests that family dysfunction is a significant concomitant of MDD and affects the course and clinical outcome of the disorder [13,14].

Grounded in Family systems theory, the Circumplex Model of Marital and Family Systems [15] gives significant insights into the intrafamilial relationships of people with mental health difficulties. According to the Circumplex Model, family cohesion and adaptability (or flexibility) are two essential elements that determine family relationships [16]. Family cohesion is described as the emotional link that family members share with one another, whereas family flexibility is concerned with the quality and expression of leadership and organization, role relationships, and relationship norms and negotiations [17].

Within the Circumplex Model, cohesion can range from disengaged (extremely low levels of cohesion) to enmeshed (extremely high levels of cohesion). In a similar vein, flexibility can range from rigid (extremely low levels of flexibility) to chaotic (extremely high levels of flexibility). Therefore, family dysfunction is characterized by values at either extreme of the spectrum, whereas optimal functioning needs balanced degrees of cohesion and flexibility (represented by mid-range values) [18]. Although family cohesion and flexibility are thought to be significant in a patient’s recovery from a severe mental disorder, these aspects of family functioning have received little attention.

### 1.3. Self-Regulation Processes in MDD

Personal beliefs about health problems have been associated with emotional and behavioral responses to those health problems and health outcomes. This area has been extensively investigated in relation to physical health [19,20] but somewhat neglected in mental health. According to the Common-Sense Self-Regulation Model [21,22], illness representations and coping are the two main processes underlying adaptation to illness in people with chronic disease.

Illness representations are structured around six interrelated dimensions: the identity dimension concerns how patients recognize their illness and which and how severe they believe their symptoms to be. Causal representations are patients’ beliefs about what contributed to the development of their disease. Consequences representations are beliefs about the effects the disease has on different areas of the patients’ lives. Timeline representations comprise patients’ beliefs about how long their illness will last and whether it is cyclical (i.e., progresses in episodes). Coherence representations are formed by patients’ perceived understanding of their diagnosis. Personal control/treatment control representations are patients’ beliefs about whether they and their treatment can affect the course of their illness. Finally, emotional representations are patients’ emotional responses to the disease (e.g., anger, anxiety, etc.). The structure of illness perception provides a framework and a proximal guide for coping behavior, with both direct and indirect effects upon health-related, psychological, social, and behavioral outcomes [20,23,24].

Although the Common-Sense Self-Regulation Model originally refers to physical illnesses, its applicability to mental disorders is supported by recent studies examining the effects of illness representations on health-related outcomes in patients with psychiatric disorders [23,24]. Illness representations have been established as significant determinants of clinical outcomes in MDD [25,26,27,28,29]. According to a recent systematic review by Mavroeides and Koutra [30], illness representations were found to be associated with a variety of clinical and treatment-related outcomes in patients with MDD, including MDD symptom severity, levels of perceived stress and anxiety, psychosocial functioning, comorbidity, and medication adherence. More specifically, research suggests that maladaptive illness representations may directly impair MDD patients’ mental health [26,28,31], while negative perceptions about MDD may impact patients’ self-management [32] and decision making about their treatment [33,34].

According to the Common-Sense Self-Regulation Model, the development of illness representations and coping are influenced by a number of factors, including personal and social/environmental ones, such as the family environment [21,22]. Given that illness representations have an important influence on health-related behaviors and actual treatment adherence, their consideration of the patient’s recovery is essential. There is a paucity of studies that explore predictive concepts in depth, and it is currently unclear as to which factors are the best long-term predictors of clinical outcomes for MDD. Previous research in the mental health area (mainly in psychosis) has unraveled various other factors involved in patients’ recovery, such as illness understanding and attributions, coping strategies, and social support [35]. This has led to the inclusion of new targets for these interventions as well as new outcome measures focusing not only on patients but also on their relatives.

### 1.4. Purpose of the Present Study

The majority of traditional Greek households place a strong emphasis on family. Despite the fact that duty and obligation to the family are of the utmost importance, coping with illness and difficulties has been highlighted to be primarily a private and family matter. In addition, self-regulation is an interpersonal as well as an intrapersonal process. In this regard, a comprehensive understanding of how people think about and thus manage their illness can only be reached by taking into account the social and family context in which patients’ perceptions have been developed.

To this end, the present study aimed to prospectively investigate the direct as well as the indirect effects of perceived family cohesion and flexibility on patients’ clinical outcomes through the mediating effect of illness representations and coping strategies. We chose to test the specified model based on previous studies suggesting that family functioning predicts various cognitions and thinking styles (e.g., rumination, cognitive distortions, illness perceptions) among community and clinical samples [36,37,38]. Moreover, our model aligns with the Common-Sense Self-Regulation Model since it postulates that illness representations mediate the association between situational and environmental stimuli (such as family dynamics), coping, and clinical outcomes [21,22].

**Figure 1:**
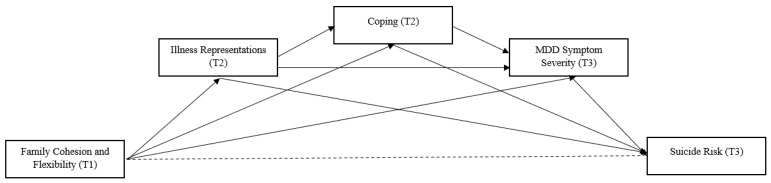
Cohesion, flexibility, illness representations, coping, symptom severity, and suicide risk in MDD: Hypothesized conceptual model.

To our knowledge, this is the first study that simultaneously examines perceived family functioning in terms of cohesion and flexibility, as well as illness representations and coping strategies, and clinical outcomes in patients with MDD. Our main hypothesis is that unbalanced levels of cohesion and flexibility influence the way patients perceive MDD (cognitive and emotional representations of MDD) and their coping strategies, which, in turn, affect their clinical outcomes (symptom severity and suicidality risk). For instance, a family system characterized by balanced levels of family cohesion and flexibility is expected to form more adaptive representations about one member’s mental illness and, consequently, more functional coping strategies to deal with stress, which may have a greater impact on reducing symptom severity and suicide risk, and the converse holds true for unbalanced levels (see Figure 1 below).

## 2. Methods

### 2.1. Sample

The present study employed a prospective design. As recommended by MacCallum et al. [39], the Root Mean Square Error of Approximation (RMSEA) was used to calculate the required sample size for our model. Our final sample size (n = 113) was marginally lower than the 117 participants required for 35 degrees of freedom, a *p* < 0.05, desired power of 0.80, RMSEA = 0 for the null hypothesis and RMSEA = 0.08 for the alternative hypothesis.

Participants were drawn from the outpatient department and the Mobile Mental Health Unit (MMHU) of the Psychiatric Clinic of the University General Hospital of Heraklion in Crete, Greece, and from an online MDD peer-support group. The following criteria were required for inclusion in the study: (i) being diagnosed with MDD by a psychiatrist using the Diagnostic and Statistical Manual of Mental Disorders (DSM-5) or the International Classification of Disease (ICD-10), (ii) having a good understanding of the Greek language, and (iii) being 18 or older. Participants were excluded from participating if they had any other severe mental illness, substance use disorder, neurological or severe physical illness, or intellectual handicap. The diagnosis of MDD was made by experienced attending psychiatrists following a clinical assessment that included the Mini International Neuropsychiatric Interview and the DSM-5 criteria [40]. Participants enrolled through the online peer support group had acquired a documented diagnosis of MDD from a psychiatrist.

**Figure 2:**
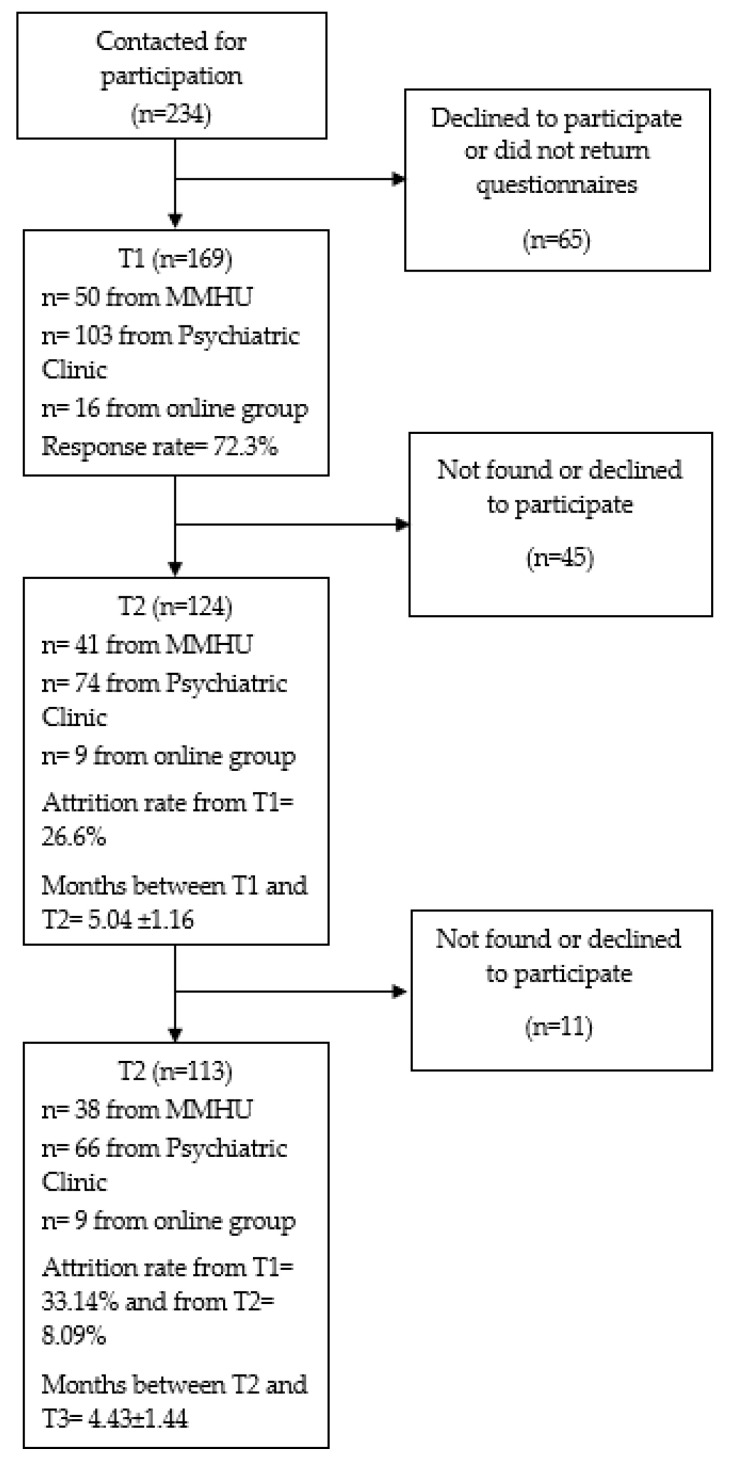
Flow diagram for study participants.

Sample size, recruitment location, mean time interval between the study’s waves, and attrition rates are presented in Figure 2. Between May 2019 and November 2020, 169 individuals with a clinical diagnosis of MDD based on a clinical interview consented to participate in the present study. Overall, 124 patients (attrition rate = 26.6%) agreed to participate and returned usable data during T2 (5.04 ± 1.16 months after the baseline assessment). A total of 113 patients participated at the T3 assessment (9.56 ± 2.52 months after the baseline assessment). The final sample included 113 patients with MDD with complete data in all study’s waves.

### 2.2. Measures

#### 2.2.1. Sociodemographic Characteristics

We used a structured questionnaire to gather information about participants’ sociodemographic characteristics, such as gender, age, educational level, marital status, employment status, family origin and current residence, financial status, etc. We also collected information about clinical characteristics, such as MDD duration and frequency of hospitalizations, as well as pharmacotherapy and psychotherapy.

#### 2.2.2. Family Adaptability and Cohesion Evaluation Scales IV Package

Perceived family functioning in terms of family cohesion and flexibility was assessed with the Family Adaptability and Cohesion Evaluation Scales IV (FACES IV) [41]. The instrument has 42 items in total and a six-factor structure, with two balanced subscales (Balanced Cohesion, i.e., Item 1, “Family members are involved in each other’s lives” and Balanced Flexibility, i.e., Item 2, “Our family tries new ways of dealing with problems”) assessing the middle range of cohesion and flexibility and four subscales (Disengaged, i.e., Item 9, “Family members seem to avoid contact with each other when at home” and Enmeshed, i.e., Item 10, “Family members feel pressured to spend most free time together” for cohesion, Rigid, i.e., Item 17, “Our family has a rule for almost every possible situation” and Chaotic, i.e., Item 18, “Things do not get done in our family” for flexibility) assessing the high and low extremes of cohesion and flexibility. The responses vary from 1, “strongly disagree”, to 5, “strongly agree.”

*Cohesion* and *Flexibility Ratio scores* were utilized to evaluate the balanced level within the family system. The ratio score is calculated by evaluating the Balanced/Average Unbalanced score for each dimension (i.e., Cohesion Ratio = Balanced Cohesion/(Disengaged + Enmeshment/2) and Flexibility Ratio = Balanced Flexibility/(Rigid + Chaotic/2). Scores ≥ 1.0 show that there is a balance of cohesion and flexibility.

The Greek adaptation of FACES IV [42] demonstrated good internal consistency (Cronbach’s alpha ranges from 0.59 to 0.79 across scales) and high test-retest reliability (intraclass correlation coefficient ranges between 0.94 and 0.97). Cronbach’s alpha values in this study ranged from 0.61 to 0.79 across scales.

#### 2.2.3. Illness Perception Questionnaire-Mental Health

The Illness Perception Questionnaire-Mental Health (IPQ-MH) [43] was used to assess patients’ illness representations. The IPQ-MH contains 67 items and includes 13 subscales: felt symptoms (i.e., Item 6, “Anxiety or fear”), symptom attribution (i.e., Item 10, “A symptom of my disorder”), timeline chronic (i.e., Item 23, “I expect to have these problems for the rest of my life”), timeline cyclical (i.e., Item 42, “My symptoms come and go in cycles”), consequences (i.e., Item 16, “My problems have major consequences on my life”), personal control (i.e., Item 30, “What I do can determine whether my problems get better or worse”), treatment control (i.e., Item 34, “My treatment can control my problems”), coherence (i.e., Item 37, “I don’t understand my problems”), and emotional representation (i.e., Item 12, “My problems worry me”) subscales, and psychological (i.e., Item 49, “Unresolved feelings resulting from the past”), biological (i.e., Item 66, “Certain genes inherited within the family”), structural (i.e., Item 47, “The lack of supportive communities”), and stress-related causes (i.e., Item 65, “The threat of an unpleasant event such as repossession of the house or redundancy”). Responses range from 1 “not at all important” to 5 “very much important” for identity representations and from 1 “strongly disagree” to 5 “strongly agree” for the remaining subscales.

To reduce the analyses’ complexity, we combined illness representations’ dimensions into broader illness schemas, which coincides with theory [22] and previous studies [44]. Hence, in accordance with our previous work [45], we grouped identity representations (symptoms experienced), consequences, chronicity, cyclicality, and emotional representations into *representations about the impact of MDD* (possible range 28–140). Moreover, coherence, personal control, and treatment control formed the dimension of *representations of control over MDD* (possible range 13–65).

The IPQ-MH has been validated in the Greek population [46]. In the present study, Cronbach’s alpha value was 0.95 for impact representations and 0.86 for control representations.

#### 2.2.4. Brief Cope Orientation to Problems Experienced

The Brief Cope Orientation to Problems Experienced (Brief COPE) [47], consisting of 28 items, was used to assess a self-report questionnaire assessing 14 different coping strategies. Participants respond to each item using a 4-point Likert-type scale that ranges from 1, “I haven’t been doing this at all”, to 4, “I’ve been doing this a lot.” In accordance with previous research [48,49], in this study, we grouped active coping, utilization of informational support, and planning into *problem-focused coping* (i.e., Item 2, “I’ve been concentrating my efforts on doing something about the situation I’m in”, possible range 6–24). Similarly, acceptance, use of emotional support, humor, positive reframing, and religious coping were grouped into *emotion-focused coping* (i.e., Item 15, “I’ve been getting comfort and understanding from someone”, possible range 10–40). Finally, behavioral disengagement, denial, self-distraction, self-blame, substance use, and venting formed the *dysfunctional/maladaptive coping* style dimension (i.e., Item 6, “I’ve been giving up trying to deal with it”, possible range 12–48).

The Brief COPE has been validated for use in the Greek population [50]. In the current study, Cronbach’s alpha values were as follows: 0.77 for problem-focused coping, 0.63 for emotion-focused coping, and 0.67 for maladaptive coping.

#### 2.2.5. Beck Depression Inventory

The Beck Depression Inventory (BDI) is a 21-item multiple-choice self-report questionnaire designed to assess MDD symptoms experienced during the past week [51]. The 21 items included reflect a variety of symptoms and attitudes commonly found among clinically depressed individuals (i.e., low mood, self-dislike, guilt, irritability, social withdrawal, sleep disturbances such as insomnia, loss of appetite, etc.). Responses to the BDI items are made on a 4-point Likert-type rating scale ranging from 0 to 3 (possible range 0–63). Higher total scores indicate more severe MDD symptoms.

The BDI has been validated for use in the Greek language [52]. Cronbach’s alpha was 0.92 in the current study.

#### 2.2.6. Risk Assessment Suicidality Scale

The Risk Assessment Suicidality Scale (RASS) [53] is a self-report questionnaire consisting of 12 items designed to evaluate suicidal risk behaviors and beliefs. Respondents provide their answers to the RASS items on a 4-point Likert–type rating scale with values that range from 0 = “Not at all” to 3 = “Very much”. The RASS covers a range of suicide risk aspects, including suicide intention (i.e., Item 6, “Do you often think of committing suicide if you have the chance?”), history of suicide attempts-non-suicidal self-injury (i.e., Item 12, “Have you ever attempted suicide during your whole life so far?”), and attitude towards life (i.e., Item 4, “Have you felt that it’s not worth living?”).

In the current study, we used a RASS total score ranging between 0 and 1190, with higher scores indicating greater suicidal risk. In the present study, Cronbach’s alpha for the total RASS score was 0.85.

### 2.3. Procedures

The present study is part of a larger prospective study assessing personal and family psychosocial determinants of MDD course. In this study, we used T1 data regarding patients’ sociodemographic characteristics and family functioning, T2 data regarding patients’ representations of MDD and coping styles, and T3 data regarding symptom severity and suicide risk.

The scales were administered to MDD patients by a trained clinical psychologist in individual sessions either in the Psychiatric Clinic of the University Hospital of Heraklion, Crete, Greece, or in Medical Health Centres in the provinces where participants were asked to take part in a study assessing psychosocial determinants (i.e., personality dimensions, early maladaptive schemas, and family functioning) and the course of MDD in Greece. The scales were also answered electronically by patients with MDD who were recruited from an online peer-support group and completed them individually. Specifically, a post about the study was made on the group’s website, along with a link to a Google Form containing the questionnaires. Individuals who were interested in participating in the study could access the survey online through the posted link.

Participants were provided with an information sheet explaining the study’s objectives. In both cases, during the initial assessment, participants were informed about the follow-up assessments, and those who wanted to take part in them provided a phone number or an email address to the researchers to contact them. The research was approved by the Research Ethics Committee of the University of Crete (registration number: 44/18 March 2019). Following a discussion of the research’s objectives and methodology, all participants gave written informed consent to participate in the study in accordance with the Helsinki Declaration [54].

### 2.4. Statistical Analysis

To investigate whether missing cases were missing completely at random, we used Little’s Test of Missing Completely at Random. Homoscedasticity was assessed by creating a plot of predicted values versus residuals. Linearity was examined using P-P plots. To evaluate normality, we used skewness and kurtosis values. Multicollinearity was tested with the use of the variance inflation factor (VIF) and tolerance and cut-off values set at 5 and 0.20, respectively [55,56]. The presence of outliers was investigated using Mahalanobis distance at a significance level of *p* < 0.001 [57].

Descriptive statistics were utilized to describe the sample’s characteristics. Specifically, we calculated means and standard deviations for continuous variables and frequencies and proportions for categorical variables. Cronbach’s alpha coefficient was used to investigate the internal consistency of the scales. Moreover, we employed the Pearson correlation coefficient (r) to assess the strength of the association between continuous dependent and independent variables. Differences in continuous variables between MDD patients and healthy controls, patients who participated in T2 and T3 and those who did not, and patients from peer support and hospital-based patients were examined using Student’s *t*-test for independent samples. Finally, correlations between family cohesion, flexibility, illness representations, coping, symptom severity, suicidality risk, and age were assessed using the Pearson correlation coefficient.

Possible control variables were identified using a series of MANOVAs. Each MANOVA included family cohesion, flexibility, illness representations, coping, symptom severity, and suicide risk as the dependent variables. Independent variables were gender (male, female), marital status (married, non-married, widowed/divorced), educational level (elementary/high school, lyceum/some years in university, university degree), employment status (working, not working), duration of MDD (<6 months, 6–12 months, 1–2 years, 3–4 years, >5 years), pharmacotherapy (no, yes), psychotherapy (no, yes), hospitalization (no, yes), and suicide attempts (no, yes). Analysis of Variance (ANOVA) and Student’s *t*-test were used as discriminant analyses to follow up the MANOVA models and identify specific group differences in the main variables.

Structural Equation Modelling was used to investigate the direct and indirect effects of family dynamics on suicide risk through illness representations, coping, and MDD symptom severity. A complete mediation model was tested by assuming only indirect effects between family dynamics and suicide risk. Bootstrapping with 5000 resamples and 95% confidence intervals (CIs) was used. CIs not containing zero were indicative of significant indirect effects. To account for the multiple aspects of the structural model fit, we evaluated the results using a range of goodness-of-fit indices, namely Chi-square (χ^2^), Goodness of Fit Index (GFI), Normed Fit Index (NFI), Incremental Fit Index (IFI), Comparative Fit Index (CFI), Standardized Root Mean Squared Residual (SRMR), and Root Mean Square Error of Approximation (RMSEA). Cutoff values were: GFI and NFI ≥ 0.90 [58], IFI and CFI ≥ 0.90 for acceptable fit and ≥0.95 for excellent fit, RMSEA ≤ 0.06, SRMR ≤ 0.08, and *p* for χ^2^ ≥ 0.05 [59].

All statistical analyses were performed using SPSS Statistics version 27 software (IBM, Armonk, NY, USA) and AMOS 27. Estimated associations are described in terms of β-coefficients (beta) and corresponding 95% CIs. All hypothesis testing was conducted assuming a 0.05 significance level and a two-sided alternative hypothesis.

## 3. Results

### 3.1. Sample Sociodemographic and Clinical Characteristics and Missing Data

Patients’ sociodemographic and clinical characteristics are presented in Table 1. The sample consisted of 19 males (16.8%) and 94 females (83.2%), ranging in age from 18 to 73 years, with a mean age of 47.11 ± 13.96 years (x ± SD). The majority of the patients were married (65.5%) and derived from and living in urban areas (56.6% and 59.3%, respectively). Regarding education, 32.7% of the sample had finished elementary or high school, 44.2% had finished lyceum or had some years in university, and 23.0% had a university degree. At the time of the assessment, 56.6% were not working, whereas 28.3% and 37.2% of the sample had no income or low income (up to 650 euros per month), respectively. The vast majority of the sample (91.2%) was living with family, partner, or others.

As far as clinical characteristics are concerned, patients had an onset of MDD between 13 and 67 years of age with a mean age of 40.53 ± 13.65 years (x ± SD), and 64.6% of the patients had an onset of illness at 5 years or longer. For hospitalizations, 15.9% of the sample had one hospitalization, and 8% had two or more. The length of longer hospitalization was up to 20 days for 13.2% of the sample and more than 20 days for 9.8%. History of suicide attempt was reported by 27.4% of participants, and 76.1% of patients were under pharmacotherapy, whereas a notable proportion of patients were additionally under psychotherapy (38.1%).

Of the total data, 0.2% of this study’s main variables were missing. Little’s Test of Missing Completely at Random was non-significant (*p* > 0.05). Thus, missing values are missing completely at random. There were no significant differences between patients who participated in T2 and T3 and those who did not participate in terms of gender, age, educational background, employment status, marital status, duration of MDD, and use of pharmacotherapy and psychotherapy. Patients recruited from online peer support were more likely to be in psychotherapy than participants recruited from psychiatric clinics or MMHUs (77.77% vs. 34.61%, two-tailed *p* = 0.026), but the two groups presented no other differences.

### 3.2. Descriptives and Bivariate Correlation between the Study Variables

Descriptive characteristics, reliability index, and intercorrelations for the study variables are presented in Table 2. Significant shared variance between the two measures of family functioning (cohesion and flexibility) was found (r = 0.75, *p* < 0.001). The two components of IPQ-MH (impact and control representations) were negatively correlated (r = −0.67, *p* < 0.001). Cohesion and flexibility were negatively correlated with impact representations, as well as maladaptive coping and suicide risk. Cohesion was also related to symptom severity. The two hypothesized mediators (impact and control representations) were significantly associated with the serial mediator variables (coping dimensions) and the outcome variable (suicide risk). Specifically, high levels of control representations were associated with increased emotion-focused and problem-focused coping and decreased maladaptive coping and suicide risk. On the contrary, high levels of impact representations were related to decreased emotion-focused and problem-focused coping and increased maladaptive coping and suicide risk. Finally, problem-focused and emotion-focused coping were linked to lower suicide risk, whereas maladaptive coping was linked to higher suicide risk.

### 3.3. Confounding Variables

Non-significant differences in family cohesion, flexibility, illness representations, coping, and MDD outcomes were found with respect to gender Wilks’ λ = 0.887, F(9, 103) = 1.45, *p*  > 0.05, partial η^2^ = 0.113, education Wilks’ λ = 0.814, F(18, 204) = 1.22, *p*  > 0.05, partial η^2^ = 0.098, duration of MDD Wilks’ λ = 0.738, F(36, 376) = 0.73, *p* > 0.05, partial η^2^ = 0.073, and hospitalizations Wilks’ λ = 0.874, F(9, 103) = 1.65, *p* > 0.05, partial η^2^ = 0.126. Significant differences in illness representations, coping, and MDD outcomes were observed with respect to marital status Wilks’ λ = 0.669, F(18, 204) = 2.52, *p* = 0.001, partial η^2^ = 0.182, working status Wilks’ λ = 0.807, F(9, 103) =  2.73, *p* = 0.007, partial η^2^ = 0.193, history of suicide attempts Wilks’ λ = 0.822, F(9, 103) = 2.48, *p* = 0.013, partial η^2^ = 0.178, pharmacotherapy Wilks’ λ = 0.836, F(9, 103) = 2.50, *p* = 0.025, partial η^2^ = 0.164, and psychotherapy Wilks’ λ  = 0.823, F(9, 103) = 2.46, *p* = 0.014, partial η^2^ = 0.177.

More specifically, married participants (*M* = 1.56, *SD* = 0.49) reported higher cohesion than unmarried ones (*M* = 1.14, *SD* = 0.51), *F*(2, 110) = 6.94, *p* = 0.002, higher flexibility (*M* = 1.30, *SD* = 0.41) vs. (*M* = 0.90, *SD* = 0.38), *F*(2, 110) = 8.31, *p* < 0.001, and lower problem-focused coping (*M* = 15.75, *SD* = 4.27) vs. (*M* = 19.19, *SD* = 2.81), *F*(2, 110) = 5.03, *p* = 0.008. Working participants (*M* = 1.60, *SD* = 0.45) reported significantly higher cohesion than non-working (*M* = 1.35, *SD* = 0.49), *t*(111) = 0.94, *p* = 0.009, higher flexibility (*M* = 1.32, *SD* = 0.41) vs. (*M* = 1.12, *SD* = 0.38), *t*(111) = 0.12, *p* = 0.012, lower depressive symptom severity (*M* = 13.32, *SD* = 11.22) vs. (*M* = 20.03, *SD* = 12.25), *t*(111) = 1.84, *p* = 0.002, and lower suicide risk (*M* = 255.41, *SD* = 244.25) vs. (*M* = 376.17, *SD* = 308.044), *t*(111) = 4.50, *p =* 0.011. Participants with a history of suicide attempts (*M* = 1.23, *SD* = 0.41) reported lower family cohesion than non-attempters (*M* = 1.55, *SD* = 0.49), *t*(111) = 3.15, *p* = 0.002, lower flexibility (*M* = 1.03, *SD* = 0.31) vs. (*M* = 1.27, *SD* = 0.42), *t*(111) = 2.83, *p* = 0.005, lower control representations (*M* = 43.58, *SD* = 7.13) vs. (*M* = 47.53, *SD* = 8.30), *t*(111) = 2.34, *p* = 0.021, higher impact representations (*M* = 108.62, *SD* = 21.87) vs. (*M* = 89.38, *SD* = 26.42), *t*(111) = −3.60, *p* < 0.001, higher maladaptive coping (*M* = 28.44, *SD* = 6.22) vs. (*M* = 25.23, *SD* = 5.24), *t*(111) = −2.75, *p* = 0.003, higher depressive symptom severity (*M* = 21.89, *SD* = 14.05) vs. (*M* = 15.31, *SD* = 11.03), *t*(111) = −2.34, *p* = 0.023, and higher suicide risk (*M* = 489.03, *SD* = 317.98) vs. (*M* = 261.34, *SD* = 249.61), *t*(111) = −3.59, *p* < 0.001. Patients receiving pharmacotherapy reported lower problem-focused coping (*M* = 15.77, *SD* = 4.40) than patients not receiving pharmacotherapy (*M* = 18.79, *SD* = 0.80), *t*(111) = 4.01, *p* < 0.001, and higher symptom severity (*M* = 18.81, *SD* = 12.56) vs. (*M* = 11.74, *SD* = 9.45), *t*(111) = −3.14, *p* = 0.003. Finally, patients on psychotherapy (*M* = 17.99, *SD* = 3.90) reported higher problem-solving coping than patients who were not receiving psychotherapy (*M* = 15.57, *SD* = 4.29), *t*(111) = −3.07, *p* = 0.003. Age was significantly and positively correlated with cohesion *r*(111) = 0.25, *p* = 0.006 and flexibility *r*(111) = 0.27, *p* = 0.004, and negatively correlated with problem-focused coping *r*(111) = −0.23, *p* = 0.014 and suicide risk *r*(111) = −0.19, *p* = 0.038. Hence, age, marital status, working status, previous suicide attempts, pharmacotherapy, and psychotherapy were used as confounding variables in the path analytical models.

### 3.4. Path Analysis

The hypothesized serial mediation model provided a very good fit to the data (χ^2^ = 34.036, df = 35, *p* = 0.051, χ^2^/df = 0.972, GFI = 0.96, NFI = 0.94, IFI = 1.002, CFI = 1.00, RMSEA = 0.000, 90% confidence intervals for RMSEA = 0.000–0.06).

According to path analysis results, family functioning dimensions were found to be associated—both directly and indirectly—with MDD clinical outcomes. Specifically, family cohesion had a significant negative direct effect on impact representations and MDD symptom severity. Furthermore, family cohesion had a significant indirect effect on maladaptive coping and on suicide risk. Family flexibility had only a significant positive direct effect on MDD symptom severity.

**Figure 3:**
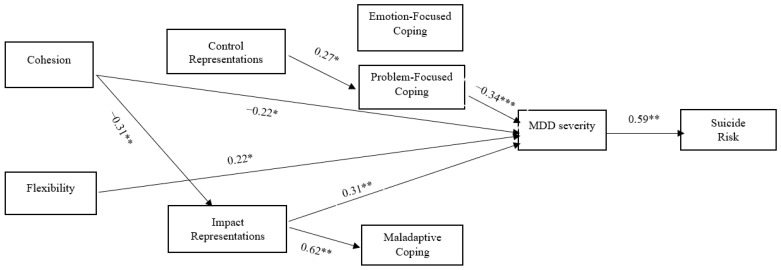
Illness representations, coping, and MDD severity serially mediate the relationship between family cohesion and MDD patients’ suicide risk: standardized path coefficients. Note. Only significant paths are presented; to reduce complexity, covariances are not depicted. Residuals of illness representations were allowed to covary. Residuals of coping styles were allowed to covary. * *p* < 0.05, ** *p* < 0.01, *** *p* < 0.001.

Additionally, impact representations had a significant positive direct effect on maladaptive coping and symptom severity and a significant indirect effect on suicide risk. Control representations had a significant positive total and direct effect on problem-focused coping. Problem-focused coping had a significant negative total and direct effect on symptom severity and a significant negative indirect effect on suicide risk. MDD symptom severity had a significant positive total and direct effect on suicide risk. Total, direct, and indirect effects are presented in Figure 3 and Table 3.

## 4. Discussion

### 4.1. Interpreting Research Findings under the Light of Literature

Combining two theories—the Circumplex Model of Family and Marital Therapy and the Common-Sense Self-Regulation Model—the purpose of this study was to examine the predictive value of a process model in which associations between family functioning in terms of cohesion and flexibility and MDD patients’ suicide risk were mediated by illness-related self-regulatory processes, as indexed by patients’ illness representations and coping strategies, as well as MDD symptom severity. To the best of our knowledge, this study is the first to examine the role of family functioning and key illness-related self-regulation processes in clinical outcomes in MDD, despite close family ties being culturally important for many Greek families, especially in Crete. The findings indicate that representations about MDD impact and symptom severity serially mediated the association between family cohesion and suicide risk in MDD. Furthermore, family cohesion was found to be linked with maladaptive coping through MDD impact representations.

In this study, we utilized composite indices of cohesion and flexibility by combining the two balanced scales of cohesion and flexibility with the four unbalanced scales of Disengaged and Enmeshed for cohesion, as well as Rigid and Chaotic for flexibility [42]. According to the Circumplex Model, balanced families with tight relationships on the one hand and organized and flexible adjustment on the other would cope better with situational stress and developmental changes throughout the family life cycle [16]. Thus, the view of the family as balanced in terms of higher degrees of cohesion and flexibility represents the family unit’s stronger ability to alter, deal with stress, or tolerate changes in family members dealing with MDD.

In our study, only family cohesion was found to contribute to an individual’s psychosocial adjustment to MDD. According to our findings, balanced levels of family cohesion were found to be related to more adaptive impact representations about MDD, that is, fewer perceived symptoms, less intense consequence and chronicity beliefs, and less intense negative emotions. It seems that a balanced level of family cohesion and harmonious family communication can convey a sense of support and security, which can help individuals ease their fear of MDD impact, thus perceiving MDD as having less detrimental consequences. In turn, this can lead to lower symptom severity and decreased suicide risk. The latter finding is consistent with studies indicating that adverse family processes may operate to increase individuals’ vulnerability to MDD or may be linked to the course and outcome of MDD, although the possible mechanisms of these relationships remain understudied [11,14].

In a similar vein, healthy levels of family cohesion were found to decrease maladaptive coping through lowering MDD impact representations. This finding is important since emerging data suggest that adaptive illness-related self-regulation processes are a protective factor in MDD [30], although the role of family functioning in the development of self-regulation skills is understudied. Maladaptive coping encompasses behavioral disengagement, denial, self-distraction, self-blame, substance use, and venting, while it has been associated with high levels of psychological distress, such as symptoms of anxiety and MDD, in various populations [60,61,62], as well as with MDD status and outcomes [47,63]. It seems that a positive family environment (i.e., open communication, low conflict, high support, and moderate affective involvement) can support healthy adjustment and self-management of individuals with MDD.

In this study, we hypothesized that MDD patients’ self-regulation may be a mechanism through which family cohesion affects clinical outcomes in MDD by applying the Common-Sense Self-Regulation Model [25,26] to understand how illness representations may impact coping strategies and, ultimately, clinical outcomes in MDD. Self-regulation refers to an individual’s ability to control their emotions, behaviors, and thoughts in order to produce desired effects or to avoid an undesired one [64,65]. Our findings supported previous research suggesting that having a thorough understanding of MDD and viewing it as a manageable condition with limited duration and symptomatology and without overwhelming consequences is associated with a better overall illness experience and outcome [25,26,27,28,29], whereas maladaptive illness representations are linked to maladaptive coping strategies, including denial, self-blame, and behavioral disengagement [32,47,66].

### 4.2. Strengths and Limitations

The present study has many methodological strengths, including its prospective three-wave design, the relatively large sample size, and the combination of family and personal characteristics, which were assessed with reliable and validated instruments for family functioning, illness representations, and coping and clinical outcomes in MDD (i.e., symptom severity and suicide risk) in a well-characterized, clinically diagnosed population.

Nevertheless, the present study also has some limitations. First, the current sample predominantly consisted of women seeking treatment for MDD symptoms in a regional area of Greece (Heraklion, Crete), and the vast majority of them were chronic cases. As a result, findings may fail to be generalized to males, individuals with MDD symptoms who have not sought treatment, individuals with recent onset MDD, or individuals in other geographic regions both within and outside Greece. Furthermore, other sociodemographic characteristics of Greek individuals with MDD (i.e., older age, living alone mainly in rural areas) found in a recent study in rural Crete [67] may also limit the generalizability of our findings.

In addition, although all individuals were diagnosed with MDD via a clinical interview by an experienced psychiatrist at baseline, all other measurements relied on patients’ self-report, which may be subject to reporter bias. Furthermore, the majority of participants presented mild to moderate MDD, so more severe cases of MDD were not evaluated in our study. Moreover, this study adopted a prospective design instead of a longitudinal design, which cannot reveal changes in variables over time. Future studies should consider using a cross-lagged panel model to analyze the mediating effect of longitudinal data.

Finally, given that MDD is frequently comorbid with other psychiatric disorders, it is important to consider the findings in light of the comorbid diagnostic limitation since the presence of MDD with personality disorders could impact our findings. Compared to patients with only MDD, these patients with co-occurring disorders are more likely to have greater symptom burden and poorer course and outcome, adding to the overall public healthcare burden.

### 4.3. Implications for Further Research and Practice

The findings of the present study have both theoretical and clinical implications. According to our results, a possible pathway for the impact of impaired family functioning on adaptation to MDD may be through patients’ representations and coping mechanisms with their mental health condition. Therefore, this study provides robust evidence that although family functioning has a detrimental effect on suicide risk in MDD, improving representations could be a viable solution to minimize symptom severity and, subsequently, the risk of suicide risk in patients. The illness perception approach puts the patient’s understanding of their illness at the center of their recovery efforts, thus offering a range of psychoeducational intervention possibilities [68]. A better understanding of patients’ illness representations and coping strategies needs further exploration, and future investigation should consider pairing patients’ and their partners’ reports of family functioning and illness representations and coping strategies to ascertain whether these reports are congruent.

The present study further supports that a comprehensive focus on both personal and environmental risk factors, such as family environment and illness representations and coping, is critical for the early prevention of suicidal risk in patients with MDD. Also, more specifically, individuals who have poor family functioning may be highly susceptible to suicidality risk, and the impaired family environment can increase patients’ risk of suicidal crisis through individual factors, such as illness representations about the impact of MDD. Based on that, patients with MDD may benefit from more drastic and holistic interventions, such as hospital admissions—where a longer stay may be suggested—combined with personal and family psychotherapy and frequent monitoring after being released home. Therefore, suicide prevention and intervention efforts for MDD patients should support an integrated approach that strengthens family functioning and promotes positive representations of their illness rather than focusing only on high-risk groups and their single risk factors [69].

## 5. Conclusions

Overall, the findings of the present study support the relationships between family functioning, illness regulation processes, and clinical outcomes in MDD. Illness representations, which have a key role in adaptation to illness, act as a link between family functioning and MDD clinical outcomes. Family functioning impacts the ways that patients perceive their health condition, which, in turn, affects their adaptation to MDD. In this context, insight into patients’ personal illness beliefs for MDD is imperative. Furthermore, how a patient perceives their illness affects the quantity and quality of the efforts they make to change their health situation as much as possible (i.e., through help-seeking and their active participation in the treatment process). Gaining knowledge on how family functioning is related to clinical outcomes in MDD and examining the underlying processes and mechanisms is crucial to inform the development of psychoeducational family-based interventions specifically designed to target unhealthy family functioning along with negative illness perceptions and dysfunctional coping and to further develop theoretical approaches.

## Figures and Tables

**Table 1:** Sociodemographic and clinical characteristics of participants (N = 113).

Sociodemographic Characteristics			Clinical Characteristics		
	N	%		N	%
**Gender**			**MDD onset**		
Male	19	16.8	<6 months	2	1.8
Female	94	83.2	6–12 months	3	2.7
**Nationality**			1–2 years	20	17.7
Greek	111	98.2	3–4 years	15	13.3
Other	2	1.8	≥5 years	73	64.6
**Origin**			**Hospitalization**		
Urban	64	56.6	None	86	76.1
Rural	49	43.4	1	18	15.9
**Residence**			2 or more	9	8.0
Urban	67	59.3	**Duration of hospitalization**		
Rural	46	40.7	Up to 10 days	4	3.5
**Marital status**			11–20 days	11	9.7
Unmarried	18	15.9	21–30 days	9	8.0
Married	74	65.5	31+ days	2	1.8
Divorced/Widowed	21	18.6	No hospitalization	87	77.0
**Education**			**Last hospitalization**		
Elementary/High school	37	32.7	Within the last 6 months	8	7.1
Lyceum/Some years in university	50	44.2	6–12 months	4	3.5
University degree	26	23.0	1–2 years	8	7.1
**Employment status**			3+ years	6	5.3
Working	49	43.4	No hospitalization	87	77.0
Not working	64	56.6	**Suicide attempt**		
**Monthly income**			Yes	31	27.4
No individual income	32	28.3	No	82	72.6
1–650 €	42	37.2	**Pharmacotherapy**		
651–1000 €	30	26.5	Yes	86	76.1
>1000 €	9	8.0	No	27	23.9
**Living**			**Psychotherapy**		
Alone	10	8.8	Yes	43	38.1
With family/partner/others	103	91.2	No	70	61.9
	**Min-Max**	**Mean (SD)**		**Min-Max**	**Mean (SD)**
**Age (years)**	18–73	47.11 (13.96)	**Age at illness’s onset**	13–67	40.53 (13.65)

**Table 2:** Descriptive characteristics, reliability indices, and intercorrelations between the study variables.

	M	SD	Skewness	Kurtosis	Cronbach’s Alpha	COH	FLEX	IR	CR	EC	PFC	MC	SS	SR
**COH (T1)**	1.46	0.49	0.02	−0.39	- ^a^	1								
**FLEX (T1)**	1.21	0.41	0.47	0.31	- ^b^	0.76 ***	1							
**IR (T2)**	94.65	26.59	−0.41	−0.32	0.95	−0.36 ***	−0.31 **	1						
**CR(T2)**	46.44	8.16	−0.00	−0.41	0.86	0.14	0.17	−0.67 ***	1					
**EFC (T2)**	26.42	5.22	0.14	−0.68	0.63	0.08	0.04	−0.21 *	0.24 **	1				
**PFC (T2)**	16.49	4.30	−0.08	−1.04	0.77	−0.03	−0.06	−0.35 ***	0.44 ***	0.53 ***	1			
**MC (T2)**	26.11	5.68	0.44	0.26	0.67	−0.25 **	−0.22 *	0.56 ***	−0.31 **	−0.02	−0.01	1		
**SS (T3)**	17.12	12.23	0.59	−0.73	0.92	−0.24 *	−0.10	0.53 ***	−0.43 ***	−0.25 **	−0.50 ***	0.29 **	1	
**SR (T3)**	323.81	287.33	1.14	0.53	0.85	−0.36 ***	−0.30 **	0.57 ***	−0.39 ***	−0.27 **	−0.27 **	0.41 ***	0.67 ***	1

Abbreviations: COH: cohesion; FLEX: flexibility; IR: impact representations; CR: control representations; EC: emotion-focused coping; PFC: problem-focused coping; MC: maladaptive coping; SR: suicide risk; SS: symptom severity. * *p* < 0.05. ** *p* < 0.01, *** *p* < 0.001. ^a^ COH indicates Cohesion Ratio. The Cronbach’s alpha values for the three subscales of FACES-IV, for which the Cohesion Ratio is calculated, are as follows: 0.79 for Balanced Cohesion, 0.71 for Disengaged, and 0.61 for Enmeshment. ^b^ FLEX indicates Flexibility Ratio. The Cronbach’s alpha values for the three subscales of FACES-IV, for which the Flexibility Ratio is calculated, are as follows: 0.75 for Balanced Flexibility, 0.66 for Rigid, and 0.63 for Chaotic.

**Table 3:** Direct, indirect, and total effects of family dynamics on MDD patients’ self-regulation processes and clinical outcomes.

	Direct Effect ^a^				Indirect Effect ^a^				Total Effect ^a^			
	β	SE	95% CI	*p*	β	SE	95% CI	*p*	β	SE	95% CI	*p*
COH-T1 → IR-T2	**−0.31**	**0.14**	**−0.56, −0.006**	**0.04**	-	-	-	-	**−0.31**	**0.14**	**−0.56, −0.006**	**0.04**
COH-T1 → CR-T2	0.03	0.15	−0.26, 0.34	0.77	-	-	-	-	0.03	0.15	−0.26, 0.34	0.77
COH-T1 → PFC-T2	0.000	0.11	−0.23, 0.23	0.99	0.08	0.08	−0.07, 0.25	0.29	0.08	0.14	−0.21, 0.36	0.57
COH-T1 → MC-T2	−0.005	0.14	−0.26, 0.27	0.99	**−0.19**	**0.08**	**−0.36, −0.02**	**0.02**	−0.19	0.12	−0.43, 0.05	0.11
COH-T1 → EFC-T2	0.08	0.15	−0.20, 0.38	0.53	0.02	0.06	−0.09, 0.16	0.63	0.11	0.14	−0.17, 0.40	0.41
COH-T1 → SS-T3	**−0.22**	**0.11**	**−0.44, −0.005**	**0.03**	−0.14	0.10	−0.34, 0.06	0.16	**−0.36**	**0.13**	**−0.61, −0.09**	**0.01**
COH-T1 → SR-T3	-	-	-	-	**−0.28**	**0.010**	**−0.47, −0.08**	**0.01**	**−0.28**	**0.10**	**−0.47, −0.08**	**0.01**
FLEX-T1 → IR-T2	−0.07	0.13	−0.33, 0.18	0.58	-	-	-	-	−0.07	0.13	−0.33, 0.18	0.57
FLEX-T1 → CR-T2	0.16	0.14	−0.11, 0.43	0.26	-	-	-	-	0.16	0.14	−0.11, 0.43	0.26
FLEX-T1 → PFC-T2	−0.12	0.12	−0.36, 0.11	0.29	0.06	0.06	−0.07, 0.20	0.36	−0.06	0.14	−0.34, 0.22	0.64
FLEX-T1 → MC-T2	−0.04	0.13	−0.30, 0.21	0.72	−0.02	0.07	−0.18, 0.12	0.71	−0.07	0.13	−0.32, 0.17	0.56
FLEX-T1 → EFC-T2	−0.11	0.12	−0.36, 0.13	0.35	0.04	0.04	−0.03, 0.14	0.25	−0.07	0.13	−0.34, 0.19	0.57
FLEX-T1 → SS-T3	**0.22**	**0.11**	**0.01, 0.46**	**0.04**	−0.01	0.09	−0.20, 0.16	0.87	0.21	0.14	−0.06, 0.48	0.12
FLEX-T1 → SR-T3	-	-	-	-	0.10	0.09	−0.09, 0.28	0.28	0.10	0.09	−0.09, 0.28	0.28
IR-T2 → PFC-T2	−0.23	0.14	−0.49, 0.06	0.11	-	-	-	-	−0.23	0.14	−0.49, 0.06	0.11
IR-T2 → MC-T2	**0.62**	**0.11**	**0.39, 0.83**	**0.000**	-	-	-	-	**0.62**	**0.11**	**0.39, 0.83**	**0.000**
IR-T2 → EFC-T2	−0.05	0.15	−0.31, 0.28	0.76	-	-	-	-	−0.05	0.15	−0.31, 0.28	0.76
IR-T2 → SS-T3	**0.31**	**0.12**	**0.06, 0.55**	**0.01**	0.11	0.08	−0.02, 0.29	0.11	**0.43**	**0.12**	**0.18, 0.66**	**0.001**
IR-T2 → SR-T3	0.17	0.09	−0.01, 0.36	0.17	**0.27**	**0.09**	**0.10, 0.47**	**0.002**	**0.44**	**0.09**	**0.25, 0.64**	**0.000**
CR-T2 → PFC-T2	**0.27**	**0.12**	**0.04, 0.52**	**0.02**	-	-	-	-	**0.27**	**0.12**	**0.04, 0.52**	**0.02**
CR-T2 → MC-T2	0.11	0.12	−0.14, 035	0.34	-	-	-	-	0.11	0.12	−0.14, 0.35	0.34
CR-T2 → EFC-T2	0.22	0.12	−0.02, 0.47	0.08	-	-	-	-	0.22	0.12	0.02, 0.47	0.08
CR-T2 → SS-T3	−0.05	0.10	−0.28, 0.14	0.57	−0.08	0.05	−0.21, 0.002	0.05	−0.14	0.11	−0.37, 0.07	0.22
CR-T2 → SR-T3	−0.03	0.08	−0.19, 0.13	0.68	−0.07	0.07	−0.23, 0.06	0.31	−0.10	0.09	−0.30, 0.07	0.23
PFC-T2 → SS-T3	**−0.34**	**0.09**	**−0.53, −0.16**	**0.000**	-	-	-	-	**−0.34**	**0.09**	**−0.53, −0.16**	**0.000**
PFC-T2 → SR-T3	0.10	0.07	−0.03, 0.25	0.14	**−0.20**	**0.06**	**−0.33, −0.09**	**0.000**	−0.09	0.09	−0.27, 0.08	0.29
MC-T2 → SS-T3	0.06	0.08	−0.11, 0.22	0.45	-	-	-	-	0.06	0.08	−0.11, 0.22	0.45
MC-T2 → SR-T3	0.05	0.07	−0.08, 0.21	0.45	0.03	0.05	−0.06, 0.14	0.43	0.09	0.08	−0.07, 0.25	0.27
EFC-T2 → SS-T3	0.003	0.08	−0.14, 0.16	0.91	-	-	-	-	0.003	0.08	−0.14, 0.16	0.91
EFC-T2 → SR-T3	−0.12	0.07	−0.26, 0.02	0.09	0.002	0.04	−0.08, 0.10	0.91	−0.12	0.08	−0.29, 0.05	0.17
SS-T3 → SR-T3	**0.59**	**0.06**	**0.45, 0.70**	**0.000**	-	-	-	-	**0.59**	**0.06**	**0.45, 0.70**	**0.000**

Abbreviations: COH: cohesion; CR: control representations; FLEX: flexibility; IR: impact representations; MC: maladaptive coping; PFC: problem-focused coping; SR: suicide risk; SS: symptom severity. ^a^ Standardized regression coefficients, corresponding bootstrapped 95% confidence intervals, and associated *p* values. Note: Bold font indicates statistically significant effects (*p* < 0.05).

## Data Availability

Data are contained within the article.
